# Updated Sniffin’ Sticks normative data based on an extended sample of 9139 subjects

**DOI:** 10.1007/s00405-018-5248-1

**Published:** 2018-12-15

**Authors:** A. Oleszkiewicz, V. A. Schriever, I. Croy, A. Hähner, Thomas Hummel

**Affiliations:** 1 Department of Otorhinolaryngology, Smell and Taste Clinic, Technische Universität Dresden, Fetscherstraße 74, 01307 Dresden, Germany; 2 Institute of Psychology, University of Wroclaw, ul. Dawida 1, 50527 Wrocław, Poland; 3 Department of Neuropediatric Medicine, Technische Universität Dresden, Fetscherstraße 74, 01307 Dresden, Germany; 4 Department of Psychotherapy and Psychosomatic Medicine, Technische Universität Dresden, Fetscherstraße 74, 01307 Dresden, Germany

**Keywords:** Olfaction, Sniffin’ Sticks, Threshold, Discrimination, Identification, Normative data

## Abstract

**Purpose:**

To provide up-to-date and detailed normative data based on a large-scale sample, increasing diagnostic validity by reference to narrow age groups as previous normative values were based upon smaller sample sizes—especially in the group of older subjects.

**Method:**

Data were obtained from 9139 healthy subjects (4928 females aged 5–96 years and 4211 males aged 5–91 years). The standard “Sniffin’ Sticks” test was applied, comprising threshold (T), discrimination (D) and identification (I) subtests, and yielding a TDI sum score.

**Results:**

Hyposmia was established at a TDI score of less than 30.75. Age-related changes were found in each domain, most pronounced for thresholds. Individuals aged 20–30 years performed best, whereas children below the age of 10 and adults above the age of 71 scored only half as well. Sex-related differences were in favor of women.

**Conclusions:**

Data provide guidance for assessing individual olfactory performance in relation to specific age groups. Significant gender and age effects were observed, with a most pronounced increase of olfactory test scores between age 5 through 20 years and a dramatic decrease at the age of 60 through 71 years.

## Introduction

The “Sniffin’ Sticks” test is a widely used tool for assessment of olfactory performance consisting of three subtests: olfactory threshold, odor discrimination and odor identification. It has been introduced over 20 years ago by Kobal et al. [1]. Since the first publication, test–retest reliability and validity have been established [2, 3] and the test has been successfully adapted across cultures, e.g., [4–6]. Both extended [7, 8] and abridged versions, with satisfying psychometric properties [9–11], have been proposed, along with modifications of the set of odors utilized [12–14].

The “Sniffin’ Sticks” battery is used in daily clinical practice as well as scientific research. Individual scores can be related to standard values for (a) normosmia (normal olfactory function), (b) hyposmia (impaired olfactory function) or (c) functional anosmia (residual or absent olfactory function). Additionally, there is the category of supersmellers, i.e., subjects with an extraordinary sense of smell. Although norms for the Sniffin’ Sticks test have already been published [15, 16], an update appeared advisable, based upon a large-scale sample comprising detailed age groups, as older subjects were underrepresented in previous studies. Furthermore, updated norms are necessary to monitor potential changes in olfactory performance caused by macro-scale environmental and social factors, e.g., pollution or dietary habits.

Here we present updated normative data for clinical and scientific quantitative assessment of olfactory performance in female and male subjects, with a threefold number of participants compared to previous studies. This large sample allowed us to bin individual results into age groups of 10 years each, for a more accurate reference of subjects’ olfactory performance to their coevals. In addition, the narrow age categories resulted in more homogeneous groups and facilitated—although cross-sectional in nature—a detailed insight into the dynamics of olfactory performance during the course of life.

## Materials and methods

Data were obtained from 9139 subjects [4928 females aged 5–96 years (*M* = 31.8, SD = 18.9) and 4211 males aged 5–91 years (*M* = 30.7, SD = 17.7)]. Among them, 3432 (37.5%) had been included in a previous study to establish normative data [15]. According to the inclusion criteria for the respective studies, all subjects were healthy and none reported histories for any olfactory disturbances.

Odors were delivered using felt-tip pens (“Sniffin’ Sticks”) of approximately 14 cm length and an inner diameter of 1.3 cm. These pens carry a tampon soaked with 4 ml of liquid odorant. For odor presentation, the cap was removed from the pen for approximately 3 s, the pen’s tip brought in front of the subject’s nose and carefully moved from left to right nostril and backwards [3].

The threshold was obtained in a three alternative forced choice paradigm (3 AFC) where subjects were repeatedly presented with triplets of pens and had to discriminate one pen containing an odorous solution from two blanks filled with the solvent. Phenylethanol (dissolved in propylene glycol) or *n*-butanol (dissolved in water) were used, with both odorants having been found equivalent in olfactory sensitivity testing: scores obtained with both are correlated [17]. The highest concentration was a 4% odor solution. Sixteen concentrations were created by stepwise diluting previous ones by 1:2. Starting with the lowest odor concentration, a staircase paradigm was used where two subsequent correct identifications of the odorous pen or one incorrect answer marked a so-called turning point, and resulted in a decrease or increase, respectively, of concentration in the next triplet. Triplets were presented at 20 s intervals. The threshold score was the mean of the last four turning points in the staircase, with the final score ranging between 1 and 16 points.

The discrimination task used the same 3 AFC logic. Two pens of any triplet contained the same odorant, while the third pen smelled differently. Subjects were asked to indicate the single pen with a different smell. Within-triplet intervals were approximately 3 s. As the odors used in this subtest were more intense, between-triplets intervals were 20–30 s. The score was the sum of correctly identified odors. Hence, the scores in this task ranged from 0 to 16 points. Importantly, subjects were blindfolded for the threshold and discrimination tasks to avoid visual identification of target pens.

Odor identification comprised common and familiar odorants (recognized by at least 75% of the population). Subjects were presented with single pens and asked to identify and label the smell, using four alternative descriptors for each pen. Between-pen intervals were approximately 20–30 s. The total score was the sum of correctly identified pens, thus subjects could score between 0 and 16 points.

The final “TDI score” was the sum of scores for Threshold, Discrimination and Identification subtests, with a range between 1 and 48 points.

### Statistical analyses

Data were analyzed by means of SPSS v. 25 software (SPSS Inc., Chicago, Ill., USA). Subjects were divided into nine age groups: (A) 5–10 years (*n* = 889); (B) 11–20 years (*n* = 1750); (C) 21–30 years (*n* = 2995); (D) 31–40 years (*n* = 1102); (E) 41–50 years (*n* = 847); (F) 51–60 years (*n* = 737); (G) 61–70 years (*n* = 464); (H) 71–80 years (*n* = 212); and (I) over 81 years (*n* = 143). Descriptive statistics were computed to establish norms based on the extended sample (Table 1). We examined the effects of sex (female vs male) and age (groups A–I) on TDI scores by means of analysis of variance (ANOVA). Further, we modelled effects of sex and age on separate subtest scores obtained for threshold, discrimination and identification scores, controlling for within-subject variance using repeated measures analysis of variance (rm-ANOVA). Pairwise comparisons were Bonferroni-corrected for multiple comparisons between the nine age groups. To provide guidance for assessing individual olfactory abilities in relation to specific age groups, we calculated the tenth percentile of TDI score for each age group.

**Table 1:** Normative values for the Sniffin’ Sticks test

	Female subjects				Male subjects				All subjects			
	THR	DIS	ID	TDI	THR	DIS	ID	TDI	THR	DIS	ID	TDI
*Age group A: 5–10* *years*												
*N*	138	76	340	21	170	93	314	33	308	169	654	54
Mean	7.59	10.83	12.16	27.13	6.95	10.35	12.10	23.99	7.24	10.57	12.13	25.21
SD	3.01	1.94	2.46	4.95	3.29	2.29	2.39	3.60	3.18	2.15	2.43	4.41
Minimum	1	6	2	18.25	1	6	4	18.5	1	6	2	18.25
Maximum	16	16	16	35.75	15.5	16	16	31.75	16	16	16	35.75
Percentiles												
5	3.25	8	7.05	18.35	2.28	7	8	19.2	2.75	7	8	19.06
10	3.95	8	9	19.25	3	8	9	19.5	3.25	8	9	19.38
25	5.19	9	11	24.5	4.25	8.5	10	20.13	5	9	11	21
50	7.5	11	12	28	6.75	10	12	23.75	7.125	11	12	25
75	9.25	12	14	30	9	12	14	26.5	9	12	14	28.81
90	11.53	13	15	34.6	11.75	13	15	29.45	11.75	13	15	30.63
95	13.05	14.15	16	35.73	13.11	14	16	31.23	13	14	16	32.69
*Age group B: 11–20* *years*												
*N*	439	316	759	229	363	231	645	155	802	547	1405	384
Mean	8.69	12.86	12.98	34.53	8.32	12.46	12.86	33.20	8.52	12.69	12.92	34.00
SD	2.61	1.83	1.87	4.03	2.86	1.96	1.81	4.25	2.73	1.90	1.84	4.17
Minimum	1	8	6	24	1	6	4	20.75	1	6	4	20.75
Maximum	16	16	16	44.5	16	16	16	45	16	16	16	45
Percentiles												
5	4.75	10	10	27.13	3.5	9	9	26.1	4.277	9	10	26.56
10	5.75	10	10	29.5	4.75	10	10.6	27.75	5.5	10	10	28.5
25	7	12	12	32.25	6.5	11	12	30.75	6.75	12	12	31.5
50	8.5	13	13	34.5	8	13	13	32.75	8.25	13	13	34
75	10.25	14	14	37.25	10.25	14	14	36.5	10.25	14	14	36.75
90	12	15	15	39.75	12.12	15	15	39.25	12	15	15	39.25
95	13.75	16	16	41.25	13.25	15	15	39.6	13.5	15	16	40.5
*Age group C: 21–30* *years*												
*N*	857	741	1523	716	649	600	1310	576	1506	1341	2833	1292
Mean	9.35	13.17	13.61	36.23	9.11	12.89	13.63	35.70	9.25	13.04	13.62	35.99
SD	3.00	1.84	1.97	4.07	2.96	1.88	1.72	4.35	2.98	1.86	1.86	4.20
Minimum	1	5	0	23	2.5	5	5	18	1	5	0	18
Maximum	16	16	16	48	16	16	16	47	16	16	16	48
Percentiles												
5	5	10	9.75	29.96	4.75	10	11	29	5	10	10	29.5
10	5.75	11	11	31	5.5	11	11	30.25	5.75	11	11	30.75
25	7.5	12	13	33.5	7	12	13	32.75	7.25	12	13	33.06
50	8.75	13	14	36	8.5	13	14	35.5	8.5	13	14	35.75
75	11.25	14	15	38.75	11	14	15	38.5	11.25	14	15	38.5
90	14	15	16	41.5	13.5	15	16	41.5	14	15	16	41.5
95	15	16	16	43.5	14.9	15	16	43	15	16	16	43.09
*Age group D: 31–40* *years*												
*N*	282	273	539	270	216	211	542	208	498	484	1081	478
Mean	9.14	12.93	13.64	35.94	8.66	12.67	13.63	35.05	8.93	12.82	13.63	35.55
SD	2.86	1.83	1.70	3.93	2.87	1.83	1.60	4.12	2.87	1.83	1.65	4.03
Minimum	1	6	2	23.5	1.75	6	5	22.25	1	6	2	22.25
Maximum	16	16	16	45.75	16	16	16	46	16	16	16	46
Percentiles												
5	4.79	10	11	29.5	4.25	10	11	27.48	4.421	10	11	28.74
10	5.75	11	12	31	4.75	10	12	29.25	5.5	10	12	30.5
25	7.5	12	13	33.5	6.5	11	13	32.76	7	12	13	33
50	9	13	14	36.25	8.5	13	14	35	8.675	13	14	35.5
75	11	14	15	38.75	10.69	14	15	37.5	10.75	14	15	38.5
90	12.5	15	15	40.48	12.15	15	15	40.5	12.5	15	15	40.5
95	15	15.3	16	42.5	14.04	15	16	41.39	14.5125	15	16	42.01
*Age group E: 41–50* *years*												
*N*	199	198	456	197	171	170	390	170	370	368	846	367
Mean	8.57	12.49	13.35	34.68	8.21	12.05	13.25	33.34	8.41	12.29	13.30	34.06
SD	2.61	1.87	1.71	4.03	3.15	2.21	1.85	5.36	2.87	2.04	1.78	4.73
Minimum	2.25	7	2	22.5	1	5	6	15.5	1	5	2	15.5
Maximum	16	16	16	44	16	16	16	44.25	16	16	16	44.25
Percentiles												
5	4.5	9	10	26.98	2.9	7.55	10	22.66	3.5	9	10	25.5
10	5.5	10	11	28.7	4.1	9	11	26.5	5	9	11	28.15
25	6.75	11	12	32.5	6.25	11	12	30.44	6.5	11	12	31.5
50	8.5	13	14	35.25	8.5	12	14	34	8.5	13	14	34.75
75	10.25	14	14	37.25	10	14	14	36.5	10	14	14	37
90	11.5	15	15	39.75	12.45	14	15	39.25	12	15	15	39.5
95	13.75	15	16	41.03	14.15	15	16	41.11	13.75	15	16	41
*Age group F: 51–60* *years*												
*N*	221	215	401	213	175	173	331	172	396	388	732	385
Mean	7.93	12.34	13.11	33.64	7.21	11.78	12.85	31.87	7.61	12.09	12.99	32.85
SD	2.96	1.70	1.77	4.11	3.08	2.12	2.05	5.16	3.03	1.92	1.90	4.69
Minimum	2.5	7	4	24	1	5	4	15	1	5	4	15
Maximum	16	16	16	45	16	16	16	44	16	16	16	45
Percentiles												
5	3.5	9	10	26.5	2.5	8	9	21.33	3	9	9	25.33
10	4.29	10	11	28.5	3.5	9	10	26	4	10	11	27.25
25	5.63	11	12	30.75	4.75	10	12	29.25	5.5	11	12	30.34
50	7.5	12	13	33.5	7.25	12	13	32.38	7.5	12	13	33
75	9.5	14	14	36.5	9	13	14	35.25	9.25	13	14	36.25
90	11.95	14	15	39.4	11	14	15	37.75	11.5	14	15	38.5
95	13.95	15	16	40.65	12.8	15	16	39.5	13.5	15	16	40.18
*Age group G: 61–70* *years*												
*N*	141	133	255	133	118	111	209	111	259	244	464	244
Mean	7.28	11.72	12.27	31.37	7.01	11.34	12.20	31.12	7.16	11.55	12.24	31.26
SD	2.80	1.92	2.24	4.39	3.08	2.31	2.55	5.23	2.93	2.11	2.38	4.78
Minimum	1	6	2	18.75	1	4	2	12	1	4	2	12
Maximum	16	16	16	43	16	16	16	44	16	16	16	44
Percentiles												
5	2.78	8	7.8	23.73	1.25	7	7	22	2.5	8	7.25	22.5
10	3.5	9	10	25.5	2.5	8	9	24	3.5	9	10	24.88
25	5.5	10.5	11	29.13	5.44	10	11	28.5	5.5	10	11	28.5
50	7.25	12	13	31.75	6.5	12	13	31.5	7	12	13	31.63
75	8.75	13	14	34.25	8.5	13	14	34.5	8.5	13	14	34.25
90	10.5	14	15	36.5	11	14	15	37.35	10.5	14	15	36.5
95	11.73	14.3	15	37.5	12.6	14.4	16	38.6	11.75	14	15	38.26
*Age group H: 71–80* *years*												
*N*	105	75	122	75	63	40	89	40	168	115	211	115
Mean	5.68	10.65	11.20	28.44	5.06	10.20	10.71	26.96	5.45	10.50	10.99	27.93
SD	2.73	2.73	2.77	5.71	3.06	2.47	2.94	6.87	2.86	2.64	2.85	6.15
Minimum	1	4	3	11	1	5	3	9	1	4	3	9
Maximum	13.5	16	16	41.25	12.5	15	16	38.5	13.5	16	16	41.25
Percentiles												
5	1.33	5.8	6	17.25	1	5.05	4	12.05	1	5.8	5	17.05
10	2.5	6	7	19.2	1	6.1	7	16.6	1.5	6	7	19.2
25	3.63	9	10	25.5	2.5	9	9	22.75	3.25	9	10	24.25
50	5.5	11	11	28.5	4.5	10.5	11	27.38	5.25	11	11	28.5
75	7.5	13	13	32.5	7.75	12	13	33.25	7.5	13	13	32.5
90	9.5	14	14	35.5	9.05	13	14	34.25	9.275	14	14	34.9
95	10.78	14.2	15	37.1	10.5	14.9	14	36.225	10.5	14.2	15	36.8
*Age group I: over 81* *years*												
*N*	106	19	107	20	33	16	36	16	139	35	143	36
Mean	4.20	8.74	8.04	24.03	3.61	8.63	9.17	22.39	4.06	8.69	8.32	23.30
SD	3.02	2.90	3.43	7.84	2.27	2.73	3.33	6.69	2.87	2.78	3.43	7.29
Minimum	1	4	2	11.25	1	4	2	13	1	4	2	11.25
Maximum	10.75	13	16	36.25	9	14	15	32.5	10.75	14	16	36.25
Percentiles												
5	1	4	2	11.29	1	4	4.55	13	1	4	2.2	11.89
10	1	5	3.8	12.1	1	4.7	5	13.7	1	5	4	13
25	1.25	6	6	17.06	1.125	7	6	15.56	1.25	6 9	6	16.69
50	3.75	9	8	26.5	3.5	8.5	9.5	21.5	3.5		8	25.75
75	6.5	11	10	28.25	5.375	10	12	28.94	6.25	11	12	28.38
90	8.65	13	13	35.98	6.9	12.6	13.3	31.45	8.5	12.4	13	32.8
95	10.5		14.6	36.25	7.6		14.15		10.25	13.2	14	36.25

Results are listed separately for sex and the nine age groups (THR olfactory thresholds, DIS odor discrimination, ID odor identification, TDI composite score as the sum of results for threshold, discrimination, and identification)

## Results

### Effects of sex and age on overall TDI score

**Fig. 1:**
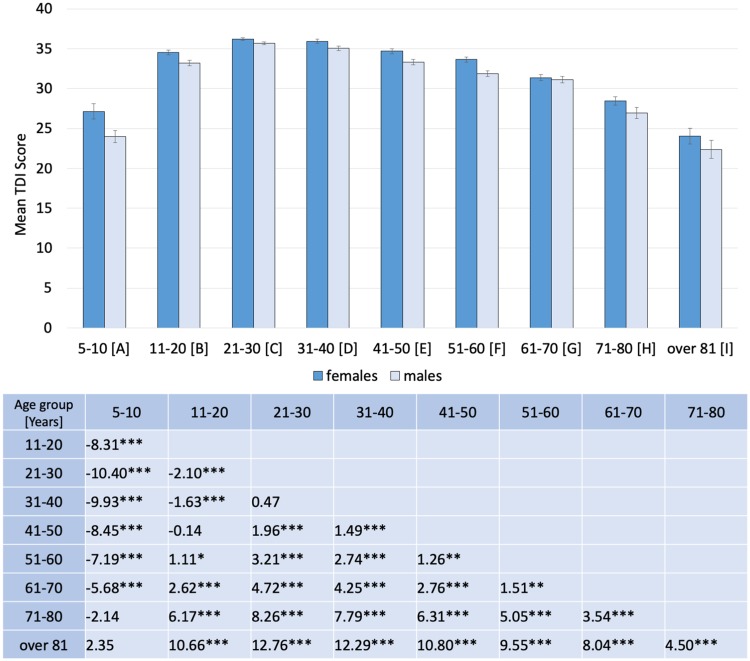
Mean TDI scores obtained from female and male subjects across the nine age groups. Error bars represent SEM. The bottom table shows differences between mean scores of two groups (group in a column − group in a row) and the level of post-hoc test significance: ***p < 0.001; **p < 0.01; *p < 0.05

**Fig. 2:**
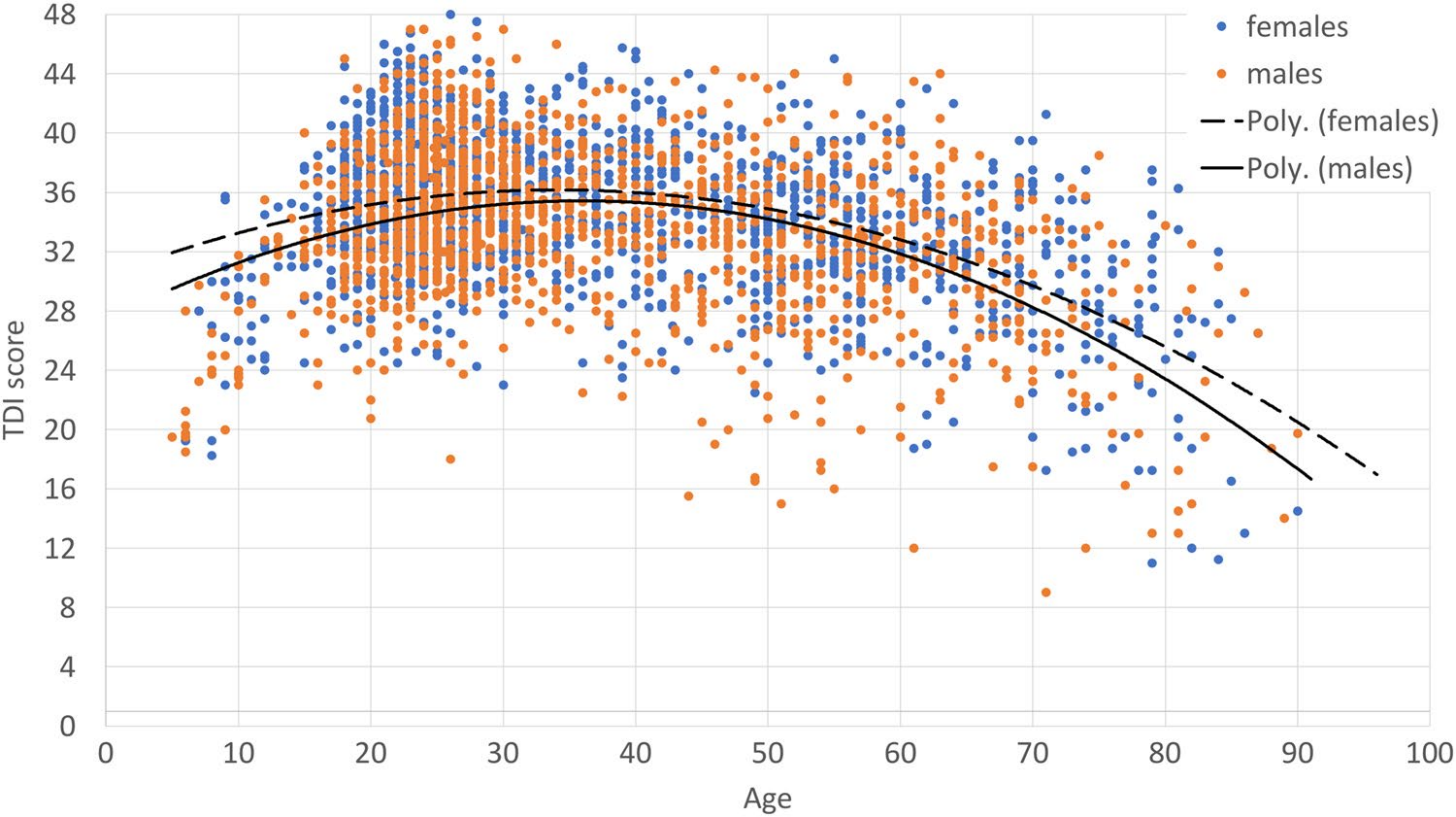
TDI scores obtained from female and male subjects with polynomial trendlines for both sexes

We found a main effect of age on the overall TDI score *F*(8, 3337) = 128.8, *p* < 0.001, *η*^2^ = 0.24. Pairwise comparisons indicated that the most pronounced increase in overall olfactory abilities occurred between group A (5–10 years) and group B (11–20 years) and the most pronounced decrease at the age of 61–70 years (Figs. 1, 2). There was also a significant yet small main effect of sex *F*(1, 3337) = 26.9, *p* < 0.001, *η*^2^ = 0.008, suggesting that on average females (*M* = 31.7 ± 0.18) outperformed males (*M* = 30.4 ± 0.19). The two factors of interest (sex and age) did not interact with each other (*p* = 0.12).

### Effects of sex and age on olfactory threshold, odor discrimination and odor identification

**Fig. 3:**
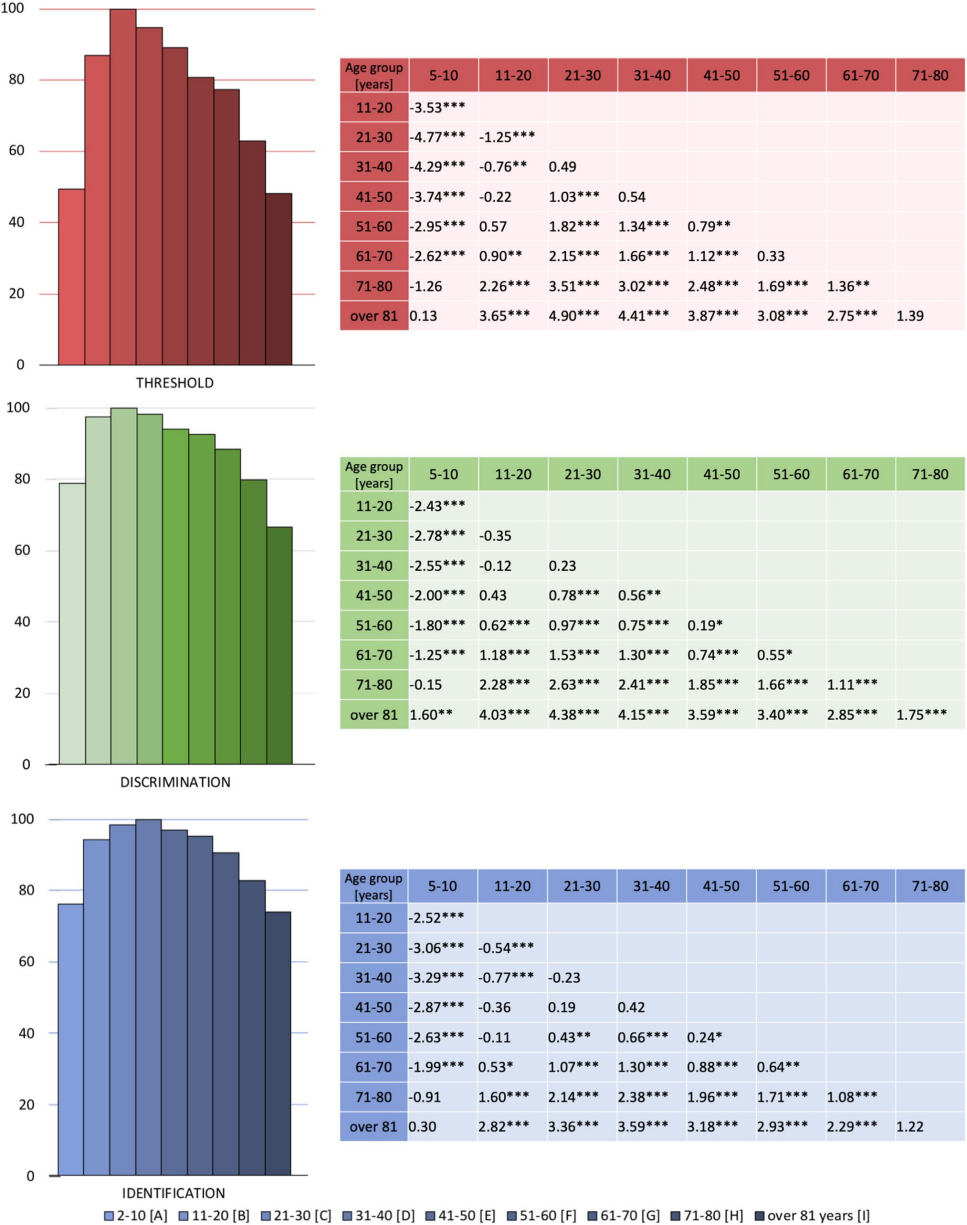
Changes of odor threshold, discrimination, and identification across the nine age groups. All data were related to the respective average results obtained in reference to the age group with highest scores (group C for threshold and discrimination; group D for identification subtest). Tables in the right panel present differences between mean scores of two groups (group in a column − group in a row) and the level of post-hoc test significance: ***p < 0.001; **p < 0.01; *p < 0.05

We observed a significant interaction between age group and subtest [*F*(16, 6646) = 7.8, *p* < 0.001, *η*^2^ = 0.02], but not between sex and subtest (*p* = 0.23). The decrease was present in each test (*F* = 128.3, *p* < 0.001, *η*^2^ = 0.24); however, it was most pronounced in the threshold task as compared to discrimination and identification. Pairwise comparisons are displayed in Fig. 3.

With pooled genders, the tenth percentile of TDI score for group A (5–10 years) was 19.4 points; group B (11–20 years) 28.5 points; group C (21–30 years) 30.75 points; group D (31–40 years) 30.5 points; group E (41–50 years) 28.15 points; group F (51–60 years) 27.25 points; group G (61–70 years) 24.88 points; group H (71–80 years) 19.2 points; group I (over 81 years) 13 points. These data provide, on one hand, guidelines for assessing individual olfactory abilities in relation to specific age groups. On the other hand, the final diagnosis of anosmia versus normosmia depends on the reference group of young adults with a cutoff value of 30.75 points.

The term “functional anosmia” refers to individuals without any or with negligible—as experienced in everyday life—sense of smell. To differentiate between “functional anosmia” and hyposmia, we established a TDI score or 16 points, which is equivalent with both identification and discrimination scores of 8, the maximum 90% of patients with anosmia would achieve, as reported earlier [16].

“Supersmellers” are subjects who reach at least the 90th percentile of the group aged 21–30 years, i.e., 41.5 or more points. Table 2 presents the proportion of subjects with functional anosmia (scoring ≤ 16 points), hyposmia (scoring between 16.25 and 30.5 points), normosmia (scoring between 30.75 and 41.25 points), and supersmellers (scoring 41.5 points or above), across the nine age groups.

**Table 2:** Percentage of participants (all of whom identified themselves as having a normal sense of smell) with normosmia, hyposmia, and functional anosmia, separately for the nine age groups

Age group (years)	% Functional anosmia	% Hyposmia	% Normosmia	% Supersmellers
5–10	0	90.7	9.3	0
11–20	0	19.5	77.1	3.4
21–30	0	9.6	79.4	11
31–40	0	10.7	83.5	5.9
41–50	0.3	20.7	75.2	3.8
51–60	0.5	28.8	68.1	2.9
61–70	0.4	38.5	59.4	1.6
71–80	3.5	60	36.5	0
Over 81	22.2	61.1	16.7	0

## Discussion

The current study provides updated norms for the “Sniffin’ Sticks” olfactory test based on a large sample. The present data obtained from 9139 subjects corroborate previous normative findings—which is noteworthy given that approximately two-thirds of the data are newly added to the database, as compared to the previous version from 2007 [15]. We observed similar values of the tenth percentile in all age groups, although the exact comparison cannot be made due to the more narrow age categories in the present study, e.g., previously, age group A was 5–15 years, whereas in the current study we present data for age groups A (5–10 years) and B (11–20 years).

With the current investigation we found the hyposmia cutoff point of 30.75 points in the reference group aged 21–30 years. Hitherto, the hyposmia cutoff score was 30.5 points [15] and in the proposed normative dataset, the same exact value of 30.5 points is the tenth percentile value of age group D (31–40 years). This 0.25 point difference between age groups C and D is likely to result from the division of the previously investigated group aged 16–35 years into two decade-wide age groups C (21–30 years) and D (31–40 years). The diagnosis of hyposmia remains to some extent an arbitrary decision, as the cutoff point of 30.75 has been established with respect to group C aged 21–30 years, representing the overall best smelling subsample. By a shift of perspective, individual scores may also be regarded in relation to the corresponding sex and age groups. We would like to give an example of how to interpret a patient’s score: A female subject aged 55 years obtained a threshold score of 4.5 points, an identification of 14 points and a discrimination score of 13 points, resulting in a TDI score of 31.5 points. According to the tenth percentile of her age group, her outcome would be “hyposmia” for the threshold test and “normosmia” for identification and discrimination. As, by definition, the more general, overall TDI sumscore overrides separate subtest results, her final diagnosis would be “normosmia”.

Importantly, changes of the tenth percentile TDI scores observed in the youngest and oldest age groups provoke the question about a deepened and updated analysis of the changes in olfactory performance [18]. Current data indicated the most pronounced loss in olfactory threshold, whereas olfactory discrimination and identification are, for one, tested with suprathreshold concentrations of odors and are, in addition, largely determined by individual experience and conscious cognitive processes which decrease at a slower pace over time. The pronounced decrease of odor thresholds with age supports the idea that it represents damage to the periphery of the olfactory system to a stronger degree than diminished odor identification and discrimination which are more strongly related to higher cognitive processes (for discussion see: [19–21]).

The relatively high percentage of children under 10 years with hyposmia is considered to be due to test difficulty rather than low olfactory function. Therefore, age-appropriate olfactory tests are necessary and have indeed been developed [9, 11, 22].

Our extended data further corroborated earlier reports on decreased olfactory abilities in age groups over 55 years [18, 23–27]. The apparent decrease in olfactory performance in seniors older than 60 raises the question about dynamics of olfactory loss with age.

“Functional anosmia”—a residual ability to perceive odors with limited usefulness in daily life—was found in a total of 0.45% of the subjects, and it was mostly prevalent in the oldest age groups, with the most visible decrease of function from age 70 years upwards. These subjects either have no olfactory function left at all or exhibit a modest ability to perceive, discriminate or identify odors insufficient for enjoyable experience of foods and drinks or the ability to detect environmental hazards such as gas, fire or food gone bad. However, age itself should not be considered a cause of olfactory loss but rather an accompanying factor of neurodegenerative diseases, drug side-effects, etc. [28, 29].

The incidence of 0.45% of participants with functional anosmia is low compared to epidemiological studies (e.g., [30]). One reason may be that the TDI score is used as the basis to establish the diagnosis of “functional anosmia”. However, using an odor identification score below eight points to determine the fraction of this population returns the number of 3.4%. It has to be considered that all subjects entering the study maintained to have a fully functional olfactory system. Yet, we found a meaningful proportion of subjects scoring in the range of hyposmia or functional anosmia, who seem either not to be aware of their olfactory dysfunction or not to be bothered by it. Finally, it must be kept in mind that the majority of the currently described population is young and healthy. Therefore no conclusions regarding epidemiology of olfactory loss in the general population can be made based on this work.

We observed sex-related differences with women outperforming men. Available empirical reports on this issue are inconclusive, with some studies pointing to a female advantage in olfactory tasks over males [15, 31, 32] but others failing to confirm this difference [33]; for review see: [34]. In our large sample, we observed the main effects of sex indicating that females obtained significantly higher scores than males—however, the difference in mean TDI scores calculated for both groups was rather small (1.3 points). In such a large sample size, even very small absolute differences become significant. In any case, the current study confirmed that sex-related differences are present but may be small; in other words, if sex-related differences are observed at all, it is typically women outperforming men.

We present updated norms for “Sniffin’ Sticks” based on a large sample of 9139 subjects. With this extended sample we found hyposmia to be defined at less than 30.75 points of TDI score in the group aged 21–30 years. Observed effects of sex and age corroborate previous norms by showing a significant decrease of olfactory abilities with age with a most pronounced increase between age 5–20 years and a most pronounced decrease at the age of 60–71 years.
